# Legion: A Platform for Gaussian Wavepacket Nonadiabatic Dynamics

**DOI:** 10.1021/acs.jctc.4c01697

**Published:** 2025-03-03

**Authors:** Rafael Souza Mattos, Saikat Mukherjee, Mario Barbatti

**Affiliations:** † Aix Marseille University, CNRS, ICR, Marseille 13397, France; ‡ Faculty of Chemistry, Nicolaus Copernicus University in Torun, Torun 87100, Poland; § Institut Universitaire de France, Paris 75231, France

## Abstract

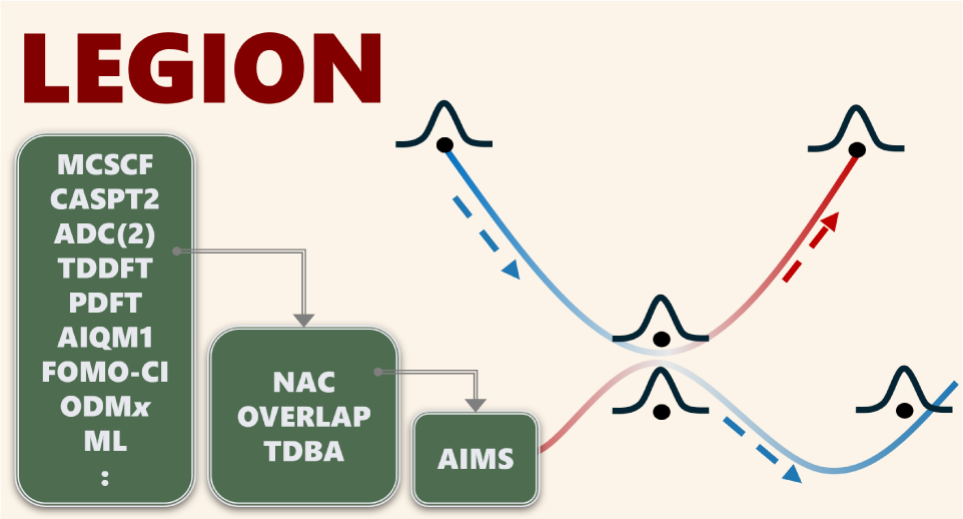

Nonadiabatic molecular dynamics is crucial in investigating the time evolution of excited states in molecular systems. Among the various methods for performing such dynamics, those employing frozen Gaussian wavepacket propagation, particularly the multiple spawning approach, offer a favorable balance between computational cost and reliability. It propagates on-the-fly trajectories used to build and propagate the nuclear wavepacket. Despite its potential, efficient, flexible, and easily accessible software for Gaussian wavepacket propagation is less common compared to other methods, such as surface hopping. To address this, we present Legion, a software that facilitates the development and application of classical-trajectory-guided quantum wavepacket methods. The version presented here already contains a highly flexible and fully functional ab initio multiple spawning implementation, with different strategies to improve efficiency. Legion is written in Python for data management and NumPy/Fortran for numerical operations. It is created under the umbrella of the Newton-X platform and inherits all of its electronic structure interfaces beyond other direct interfaces. It also contains new approximations that allow it to circumvent the computation of the nonadiabatic coupling, extending the electronic structure methods that can be used for multiple spawning dynamics. We test, validate, and demonstrate Legion’s functionalities through multiple spawning dynamics of fulvene (CASSCF and CASPT2) and DMABN (TDDFT).

## 1 Introduction

Studying deactivation paths and the lifetime of electronically excited molecules is a fundamental aspect of theoretical photochemistry. It can pave the way for multiple applications^1−4^ and has been the focus of the field of nonadiabatic dynamics.^5−7^ Of the available methods to perform this type of dynamics, multiconfiguration time-dependent Hartree (MCTDH)^8,9^ is likely the most accurate and falls within the class of quantum dynamics. The potential energy surface is precomputed, and a model system represents the molecule under study. While this method is considered the reference when the data is available, it scales poorly with the number of degrees of freedom and demands prior knowledge of the system. Novel approaches can alleviate the computational cost of running such simulations, specifically the multilayer (ML)-MCTDH,^10,11^ which groups multiple degrees of freedom into a single basis. However, the method is still reasonably limited by system size.

In an attempt to obtain more straightforward methods and software, nonadiabatic mixed quantum-classical methods^5^ were developed, such as trajectory surface hopping^12,13^ and Ehrenfest^14−16^ families of methods. Those consider classical trajectories moving independently from one another, where the swarm of trajectories will behave as what we could call a *classical wavepacket*.^a^ While those usually show good agreement with higher-level methods, they use multiple approximations and, eventually, some *ad hoc* ones.^18−20^

**Figure 1:**
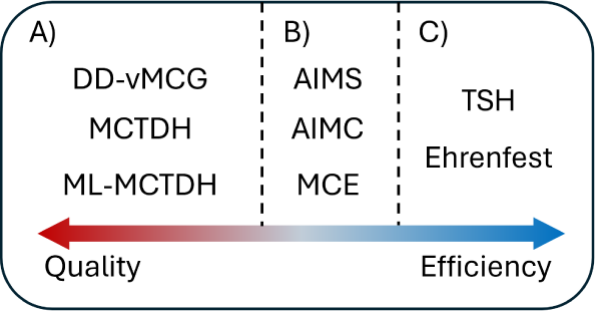
Expected qualitative placement of available nonadiabatic dynamics methods in the balance accuracy vs computational efficiency. A) Quantum wavepacket methods; B) classical-trajectory-guided quantum wavepacket methods; C) classical wavepacket methods.

Somewhere in between the high accuracy of quantum wavepacket dynamics and the computational efficiency of classical wavepackets, another family of methods emerges: the classical-trajectory-guided quantum wavepacket dynamics (Figure 1). Those propagate a swarm of classical trajectories, which are then used to construct nuclear quantum wavepackets that are evolved following the time-dependent Schrödinger equation (TDSE). In his seminal paper, Heller introduced the idea of propagating a quantum wavepacket built from a linear combination of frozen Gaussians^21^ to allow the quantum propagation of the nuclear degrees of freedom by following classical trajectories. An advantage the Gaussian wavepacket methods offer compared to classical wavepacket methods is that the quality of the result can be, in principle, systematically improved until convergence by increasing the number of classical trajectories.

To optimize the balance of quality and computational efficiency, the family of multiple spawning^22^ methods automatically expands the nuclear basis of trajectories when needed. The dynamics start with the minimum number of trajectories, but the nuclear basis is expanded in the regions of high nonadiabatic coupling by spawning new trajectories in the coupled electronic state. This scheme allows for a minimal number of trajectories to be propagated while still describing the population transfer between electronic states. Initially, the Full Multiple Spawning (FMS)^23,24^ used the idea of Gaussian propagation and presented the concept of spawning, which, in the limit of infinite trajectories, is supposed to return exact dynamics.^25^ Then, the independent first-generation approximation (IFGA) and the saddle point approximation (SPA) were introduced to allow it to work with on-the-fly electronic structure calculation and was called *Ab Initio* Multiple Spawning (AIMS).^22,24^ Multiple spawning is still being actively developed, with methods to reduce the computational cost by reducing the number of trajectories being propagated,^26,27^ and extension to incorporate intersystem crossing effects.^28−30^

Other methods have also successfully adapted the Gaussian wavepacket propagation. Multiconfigurational Ehrenfest (MCE),^15,31,32^ builds a Gaussian wavepacket over a swarm of classical trajectories, with the difference that those trajectories follow an effective potential energy surface, averaged over the adiabatic potentials.^14^ Ab initio multiple cloning (AIMC)^33,34^ also follows Ehrenfest trajectories, but at regions of high mixing between states, the trajectories are cloned in a single state each. This attempts to improve the wavepacket description at regions of high mixing^33^ while adaptively changing the nuclear basis size, as AIMS. Another Gaussian wavepacket method is the variational Multi-Configuration Gaussian (vMCG) and their direct dynamics variant (DD-vMCG),^6,35−37^ available in the QUANTICS package.^38^ This method can be classified as being closer to MCTDH, with the difference that it also follows trajectories, avoiding the restrictive limitation of grid-based methods. Different from the other Gaussian wavepacket methods mentioned, vMCG follows quantum trajectories,^36^ meaning that the Gaussian parameters (positions and widths) are propagated with equations of motion obtained from the application of the variational principle.

While the present implementation operates within the adiabatic representation, we acknowledge the challenges inherent to this framework, such as nonintegrability and double-valued boundary conditions that Gaussian basis functions cannot fully capture, as discussed in refs.^39,40^ These issues—which are partially compensated in AIMS and SH methods^41^—have motivated alternative approaches. For example, vMCG methods address these challenges by means of quasi-diabatization,^37^ which has also been suggested as a modified AIMS.^42^ Other solutions using time-dependent diabats^43,44^ and adapted nuclear basis^45^ have also been attempted. While these solutions lie outside the scope of this work, they provide valuable perspectives for future developments.

This work presents the newly developed Legion, a software for Gaussian wavepacket nonadiabatic dynamics simulation created within the Newton-X platform.^46^ Legion is intended to be a modular program that allows for the easy implementation and testing of new methods and approximations, especially in the family of classical-trajectory-guided quantum wavepacket methods. The software is written as a combination of Python for data management and Fortran for numerical operations. It is interfaced with multiple widely used electronic structure software, with some interfaced directly and others being intermediated by Newton-X.

This first Legion version is dedicated to AIMS. However, as we shall discuss, its architecture is being planned to allow direct implementation of other classical-trajectory-guided quantum wavepacket methods. When compared to trajectory surface hopping (TSH),^46−62^ we noticed fewer easily accessible open-source and efficient multiple spawning software^63−65^ with the noticeable exception of Pyspawn.^64,66^ Also, the options of electronic structure interfaces to run AIMS simulations are much more limited. Legion improves these two aspects, exploiting the computational flexibility built in Newton-X for TSH. Thus, any method or interface currently available (or to be implemented) in Newton-X automatically allows AIMS propagation with Legion. These interfaces span from semiempirical methods and machine learning potentials to CASPT2 and MRCI. Moreover, Legion has a flexible approach to nonadiabatic couplings, where even methods without explicit coupling vectors or wave functions can be employed.

## 2 Theory

### 2.1 Frozen Gaussian Propagation

In the Gaussian wave function propagation, as proposed by Heller,^21^ the molecular wave function follows the Born-Huang representation:^67^
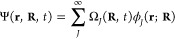
1

Here, the molecular wave function Ψ is a function of the electronic coordinates **r**, nuclear coordinates **R**, and time. It is composed of a linear combination of the electronic functions *ϕ*_*J*_, which are eigenfunctions of the electronic time-independent Schrödinger equation (TISE), and the nuclear functions Ω*_J_*. The nuclear wave functions are
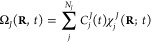
2where *C*_*j*_^*J*^ are coefficients to be determined, and χ_j_^J^ are Gaussian functions *j* associated with the electronic state *J*, centered around the positions (*R*_*j*_) and momenta (*P*_*j*_):
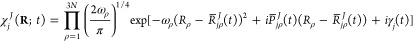
3

The index ρ runs over all nuclear coordinates. During the propagation, the widths (*ω*_*ρ*_) are specific for each atom type. They are kept constant,^68^ characterizing them as frozen Gaussians. The real-valued phase factor *γ*_*j*_ is specific for each trajectory. Each trajectory is propagated classically, independent from the others, but the coefficients **C** connect them. When the Born-Huang wave function in eq 1 is inserted into the TDSE
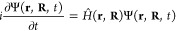
4we have
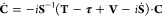
5where
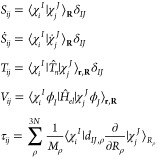
6

The matrix **S** contains information on the overlap between different Gaussians, **Ṡ** is the product of one Gaussian with the time derivative of another, **T** is the nuclear kinetic energy, **V** is the potential energy built from the electronic Hamiltonian Ĥ*_el_*, and **τ** has information on the nonadiabatic coupling, which connects trajectories in different electronic states using **d***_IJ_* = ⟨*ϕ*_*I*_|∇_R_|*ϕ*_*J*_⟩_r_.^69^ The index **r** denotes integration over the electronic coordinates, while the index **R** denotes integration over the nuclear coordinates. The Supporting Information shows the derivation of eq 5 and the functions for the matrix elements in eq 6.

The phase is independent of the coordinates and is associated with each trajectory, making it redundant with the coefficient.^64,70^ This implies that the phase is not required, being set to zero by Fedorov et al.^64^ Despite being redundant, the phase factor can still be used to improve the stability of the coefficient integration. Here, we choose phase propagation^71^ so that the amplitudes remain constant when the overlap with all other trajectories is null instead of oscillating the weight between the real and imaginary parts. This means that the diagonal elements of the resulting matrix multiplying the coefficients on the right side of eq 5 are zero, and their variation is only due to population transfer between trajectories:
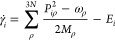
7

Where *E*_*i*_ is the electronic energy of the current state (*I*) of the trajectory *i*. The Gaussian propagation scheme is very flexible, and different methods can use the same set of equations but vary in trajectory management. In DD-vMCG,^36,72−74^ the trajectories are propagated with information from the whole nuclear basis, following a *quantum trajectory*,^36^ and the widths are also propagated; in MCE,^15,31,32^ the trajectories follow an effective Hamiltonian that averages different adiabatic electronic surfaces; in AIMC^33,34^ the trajectories also follow this effective Hamiltonian, with the modification that the nuclear basis is expanded by means of cloning. In this work, we decided to start the development with AIMS, where the trajectories follow a classical path defined by the adiabatic energy and the nuclear basis is expanded by spawning new trajectories. The basis control is separated from the Gaussian propagation, so other dynamics methods can be added without much modification to the code.

### 2.2 *Ab Initio* Multiple Spawning

**Figure 2:**
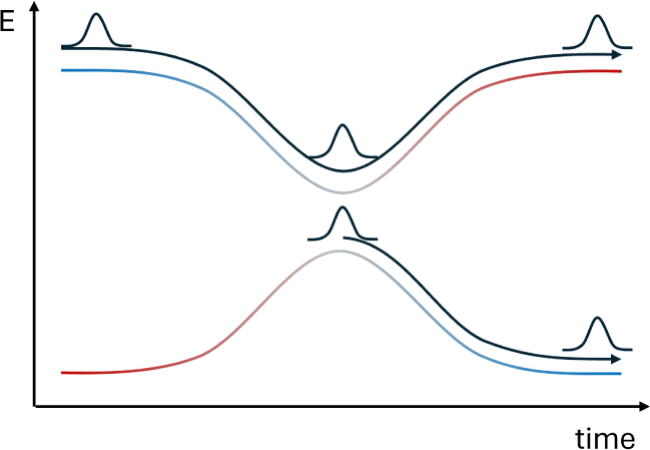
Scheme of the multiple spawning method. Classical trajectories follow the Born–Oppenheimer PES, and new trajectories are added in regions with high coupling between states.

To deal with the automatic control of the number of Trajectory Basis Functions (TBFs), trajectory spawning and elimination are employed, following predefined criteria (Figure 2). For the proper description of the interaction between different electronic states, the most critical region is where there are strong nonadiabatic couplings. To evaluate this, multiple spawning algorithms usually follow each classical trajectory, checking the norm of the nonadiabatic coupling (**d***_IJ_*) or the time derivative coupling (TDC)—dot product of velocity with the nonadiabatic coupling (**v·d***_IJ_*). Once the trajectory being followed reaches a threshold that defines the beginning of the coupling region, this trajectory is propagated independently from the rest of the system until it reaches the maximum coupling value. At this point, a child trajectory is created in the corresponding electronic state, retaining positions, energies, gradients, and coupling of the parent but adjusting the velocity to conserve the total energy between parent and child. This adjustment can follow the same relation used in the fewest switches surface hopping:
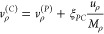
8where *v*_*ρ*_^(P)^ are the coordinates of the velocity vector of the parent trajectory and *v*_*ρ*_^(*C*)^ are the same for the child trajectory. ξ is an optimization parameter chosen so that the variation of kinetic energy between parent and child trajectories compensates for the difference in potential energies, keeping the total energy constant. *u*_*ρ*_ is an unitary vector of the direction of adjustment, that usually follows either the nonadiabatic coupling vector or the momentum direction. The derivation of the value for ξ can be found in eq S6 of ref.,^75^ called *γ*_*LJ*_ there.

The child trajectory is added to the nuclear basis and backpropagated to the origin of the coupling region. The new trajectory allows population transfer between states when the coefficients are propagated. Along a single simulation, the system should enter multiple coupling regions. As the number of trajectories increases, more will pass through new coupling regions and cause new spawns. This exponential increase in the number of TBFs can become a computational problem due to the possible cost of running multiple electronic structure calculations for each time step.

An optional strategy that can be employed to alleviate the increase of the nuclear basis is trajectory elimination, where various factors can be checked to decide whether a trajectory is still important for propagation. First, one checks whether the trajectories are coupled through the Hamiltonian in the same sense as in Energy Stochastic Selection AIMS (ESSAIMS).^26^ This observes both direct and indirect coupling, and one trajectory can only be considered for elimination if it is entirely uncoupled from all others. The second checks whether the trajectories significantly overlap with any other in the basis. If the absolute value of the overlap of the trajectory being checked with all others is smaller than a given threshold, then it can be considered for elimination. Lastly, the nuclear population of that trajectory can be computed as
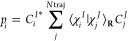
9

If the nuclear population is below a given threshold, then it is another approval for elimination. A TBF should only be removed from the basis if it passes all those checks for elimination repeatedly for a given amount of time. When the TBF is removed from the basis, the nuclear wavepacket is projected into the new reduced basis to adjust the coefficients by
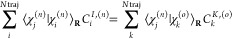
10

The new coefficients *C*_*i*_^*I*,(*n*)^ are obtained by solving the projection of the molecular wave function before elimination  into the new basis {*χ*_*j*_^(*n*)^}.

During the propagation or afterward, the expectation value of any arbitrary operator (*Ô*) can be computed for each independent simulation by the relation:^76^
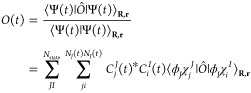
11

Under the IFGA, the total property computed with AIMS is obtained by performing a simple average of the expectation values for each simulation starting from independent initial conditions.

### 2.3 BAT Approximation

Another source of computational time is the extra electronic structure calculations that have to be performed at the centroid between pairs of trajectories. In principle, the integral presented for the potential energy (*V*_*ij*_) and the nonadiabatic (*τ*_*ij*_) terms from eq 6 run over the whole position space. SPA^25^ allows AIMS to be used in on-the-fly dynamics, approximating the integrals by the evaluation of the Gaussian, using the energies and nonadiabatic coupling at the centroid between trajectories **R̅** as in the relation:
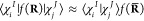
12

From here on, we are omitting the integral indexes **R** and **r**; all integrals are assumed to be over the nuclear position unless otherwise specified.

The first step to alleviate the computational effort is to perform the single-point calculation only when the centroid values are actually going to be necessary. Since all matrix elements depend on the overlap, there is no need to perform electronic structure calculations when this overlap is small enough.

Further than that, one can opt to use the bra–ket averaged Taylor expansion^33^ (BAT) to approximate those integrals without calculating the values at the centroid points. For potential energy, where the energy gradients are also available, the first-order Taylor expansion expressed in eq 13 can be used, which already considers that the potential energy term is only nonzero when both trajectories evaluated are in the same electronic state.
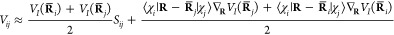
13

Similarly, we can use the zeroth-order Taylor expansion to approximate the nonadiabatic matrix element:
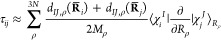
14

The BAT approximation has shown promising results^33^ while significantly reducing the amount of necessary electronic structure computations.

### 2.4 BATE—Modified BAT Approximation

We also propose a modification to the bra-ket averaged Taylor approximation that we believe has not been published before. A zeroth-order Taylor expansion approximates the centroid value for the NAC and, consequently, the TDC. Within this approximation, those curves are assumed to behave as straight lines, but the nonadiabatic coupling is known for its fast variation around a small region of the phase space, being much more similar to an exponential function, where *A* and *B* are the fitted constants, and *x* is the free variable:
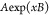
15

Under that assumption, one can use the same information used in the standard BAT to fit an exponential that passes through the values of the NAC curve at both trajectories, *i* at *x = 0* and *j* at *x = 1,* and ρ runs through the coordinates:
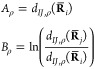
16

Then, knowing that the centroid is found in the middle of those two points, the centroid value can be computed at *x = 0.5*:
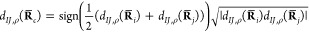
17

The same relation can be used to compute the centroid TDC when the NACs are not used. In the results, this is shown as the bra-ket averaged Taylor/exponential (BATE) approximation. To keep the values at the centroid real in eq 17, we take the square root of the absolute value of the product at different positions. The final sign is defined by the average of the two coupling vector elements.

### 2.5 Time Derivative Coupling

The requirement to use nonadiabatic coupling can be a limiting factor in propagating nonadiabatic dynamics. Various electronic structure methods can compute energy and gradients, but coupling vectors are not always available due to fundamental restrictions or implementation difficulties.^77−79^ Computing time-derivative coupling without nonadiabatic coupling is a common practice in surface-hopping methods^80,81^ and has been proposed and used for multiple spawning.^64,82,83^

The modification of the working equations is straightforward, arriving from a simple application of the chain rule in the formula for the nonadiabatic (*τ*_*ij*_) matrix elements. In the context of the SPA approximation, they can be computed with eq 18 evaluated at the centroid:
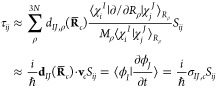
18

Meanwhile, the BAT approximation can be written as eq 19, evaluated at both trajectories:
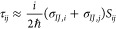
19

For the evaluation of the time derivative coupling (*σ*_*IJ*_), a method commonly used in surface hopping is based on the work of Hammes-Schiffer and Tully,^84^ which uses the overlap of the electronic wave functions at sequenced time steps
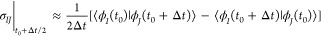
20

Alternatively, a newer proposition that uses a unitary formalism, the norm-preserving interpolation (NPI), shows potentially improved stability^82^ but also depends on the overlap between electronic wave functions:
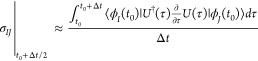
21

We are currently implementing the Hammes-Schiffer scheme as in eq 20, and we plan to add the NPI in the near future (eq 21).

#### 2.5.1 Time-Dependent Baeck-An Coupling

An alternative that circumvents the computation of the electronic overlaps entirely and does not require information about the electronic wave function has been gaining traction in surface hopping, the Baeck-An coupling,^85^ proposed independently by do Casal et al.^86^ and Shu et al.^87^ In this approach, the time derivative coupling is approximated by the relation:
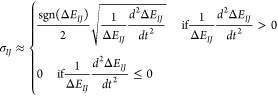
22

While the second-order central difference can approximate the energy derivative:
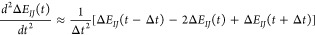
23

This method has shown good agreement with dynamics using the nonadiabatic coupling in surface hopping.^75,88,89^ To the best of our knowledge, this is the first time this method has been proposed in the context of multiple spawning, and it will extend the number of electronic structure methods available for AIMS.

## 3 Technical Aspects of the Program

### 3.1 Legion in a Nutshell

**Figure 3:**
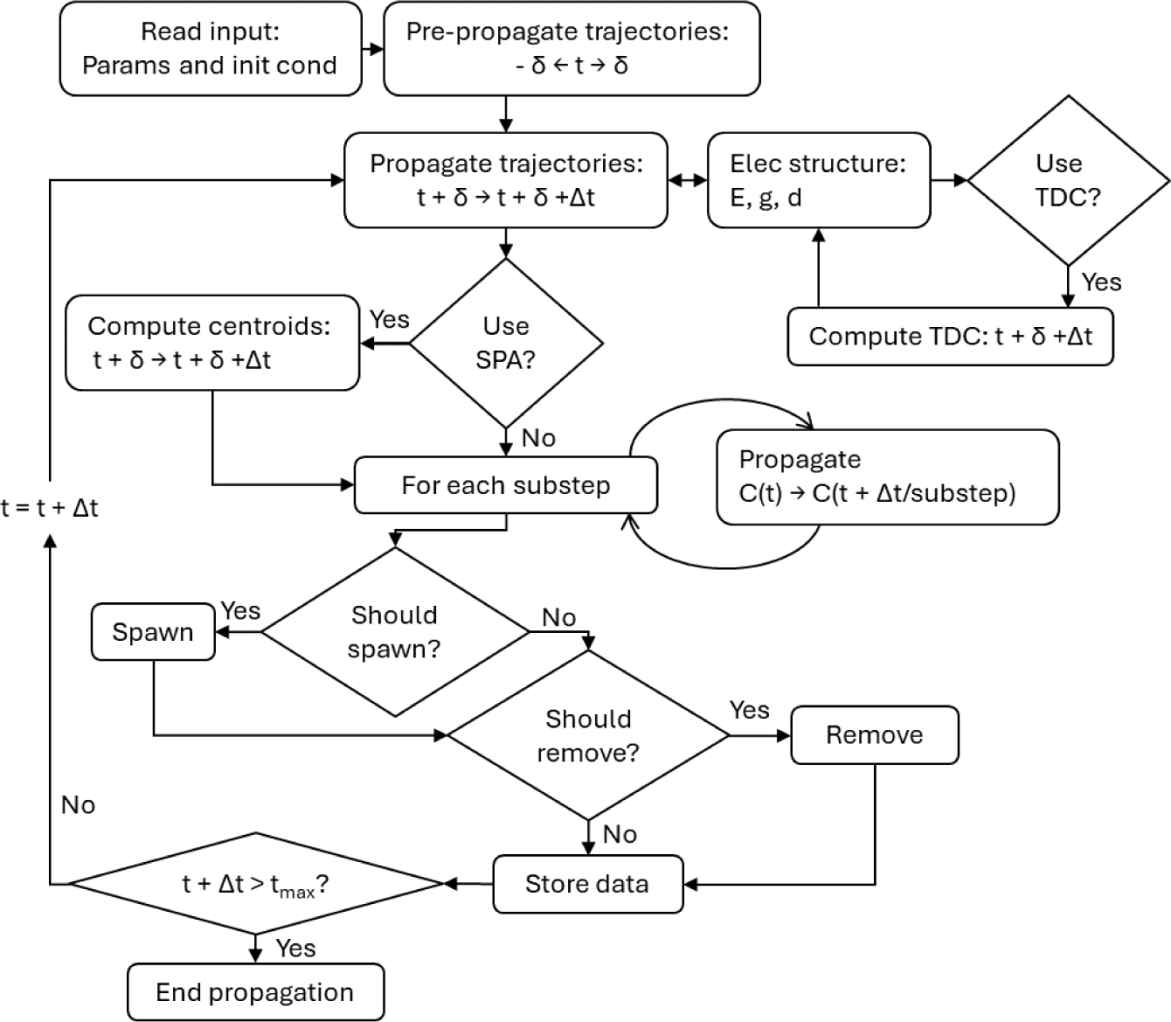
Flowchart of Legion. δ is the number of steps that the classical trajectories are propagated ahead of the coefficient, useful for the TDBA and interpolation approximations. Before starting the dynamics, the pre-propagation ensures that there will be enough points since time *t = 0* to compute the centroid numerical derivatives and interpolation.

Legion comprises independent building blocks that can be customized depending on the method intended. In the initialization, the appropriate objects will be loaded according to the parameters defined in the input file, and they can perform their task independently of the other modules:

- *Ensemble*: It aggregates the classical trajectories and the simulation information, like the overlaps and Hamiltonians used for coefficient propagation. Not only does it store the information, but it is the interface that creates new trajectories and their centroids or removes them from the simulation;
- *Nuclear Interface*: It indicates how the Hamiltonian and the forces will be computed for the trajectory propagation. For AIMS, the Hamiltonian is the one presented in eq 6, and the force is merely the negative of the gradient of the current state of the trajectory. Other methods can be easily added to the interface with little to no modification in the rest of the code;
- *Time Derivative Coupling*: If the nonadiabatic coupling vector is not read from the electronic structure, this is going to be responsible for computing the time derivative coupling using the alternative methods based on the overlap (eq 20) or the Baeck-An coupling (eq 22);
- *Integrator*: It is responsible for performing the coefficient integration using one of the methods implemented. Various methods are available, including Runge–Kutta integration, Hamiltonian diagonalization, and Magnus expansion, which contain different orders and adaptive step sizes to ensure convergence. In this case, the intermediary points are not computed with electronic structure calculations but interpolated with a cubic Hermite spline interpolation;^90,91^
- *Coefficient Integration*: It is the interface between the trajectory information and the integrator. It computes the Hamiltonians required for the integrator and creates the interpolation that the integrator will use;
- *Spawning*: It is responsible for keeping track of which trajectories should be spawning, and in the positive case, creates the new trajectory to add to the nuclear basis. It performs the propagation of the parent trajectory up to the maximum coupling point, creation of the child with energy conservation and momentum correction in the nonadiabatic coupling vector or momentum direction, and backpropagation of the child;
- *Dynamics*: This module combines the previous ones, calling each one when necessary. It contains the initialization of the dynamics and the propagation up until the maximum time, as described in Figure 3.

In some situations, running the trajectory propagation with some time advantage in relation to the coefficient integration might be interesting. For example, suppose one is computing the time derivative coupling using the TDBA approach. In that case, it is better to calculate the numerical second derivative of energy if one has information on the energy at a future time step, as seen in eq 23. In this case, Legion will automatically propagate the TBF one step ahead of the coefficients. This is possible because trajectory propagation is not dependent on the coefficients; they are only integrated synchronously so that the information on the population can be used when choosing to remove trajectories from the nuclear basis.

To perform frozen Gaussian propagation, the Gaussian width has to be chosen for each atom. Legion can read the widths from the geometry file, and one can choose from values recommended in the literature.^68,92^ Those values are restricted to only a handful of elements. In recent work,^93^ we fitted a function to obtain the Gaussian width for any element in the periodic table, computed from their atomic radius. It allows multiple spawning to be used with any element, and it delivers the default values used in Legion. The fitted formula is
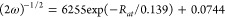
24where ω is the Gaussian width, and *R*_*at*_ is the atomic radius from ref.^94^

The initial condition necessary to start the dynamics propagation consists of two pieces of information: the initial geometry and velocity of the molecule at time zero. Legion is a software for performing the propagation of the dynamics, and for now, it does not contain a method to generate the initial condition. The Newton-X CS within the Newton-X platform provides their generation to the user. This uses the nuclear ensemble approach (NEA)^95,96^ to generate initial conditions and spectrum, usually employing the harmonic Wigner probability distribution function to generate the geometries and velocities.^97^

### 3.2 Legion in Depth

#### 3.2.1 Adaptive Time Step

A feature that improves efficiency is the capability to propagate the classical trajectories with fewer single-point calculations far from the coupling region while reducing the classical step size within this region, where the gradients and nonadiabatic coupling can vary fast. The implementation is inspired by the idea of substeps used for surface hopping.^46,98^ In that case, the time step used for integrating the coefficients has to be smaller than the time step for the classical propagation, so intermediate values are interpolated and used for coefficient propagation. Far from the coupling region, the systems are well-behaved, and the usual time step of 0.5 fs should be enough to integrate the classical trajectories smoothly. Within the coupling region, the nonadiabatic coupling varies fast, and a smaller integration step will be able to capture it better.

In Legion, the user defines the number of substeps to compute and the total time step of the dynamic. During the propagation of the classical trajectories, if they are below the coupling threshold for all states, the classical integration of the nuclei takes the full time step. The substep points are interpolated using the cubic Hermite spline interpolation.
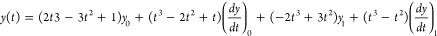
25

In it, *y*(*t*) is the value to be interpolated, *y*_*0*_ is the value on the original trajectory at the beginning of the time step, and *y*_*1*_ is the value at the end of the step. *dy/dt* are their derivatives at those same beginning and end of the time step. The free variable *t* is normalized so that *y*_*0*_*= y(0)* and *y*_*1*_*= y(1)*. The spline interpolation ensures that the points and first derivatives of the exact function and the interpolated one are the same at the extremes of the interpolation interval. Not all properties will have a derivative available, so they can be computed using the numerical derivatives in all cases.
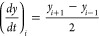
26

This again uses trajectories being propagated ahead of the coefficient to use centroid derivatives. If the classical trajectories have crossed the coupling threshold, using the same criteria checked for spawning, then instead of interpolation, the time step is reduced to the size of the substep, and no interpolation is necessary.

The coefficient integration is unaware of this process and computes the new coefficients for each substep. This way, part of the trajectories may be within the coupling region and being propagated every substep, while the other part is taking larger steps and complementing the data with interpolation.

#### 3.2.2 Electronic Structure Interfaces

Legion requires an external electronic structure program to propagate the classical trajectories. This program is responsible for computing the energies, gradients, and, optionally, nonadiabatic couplings. Living under the umbrella of the Newton-X platform, Legion inherits all the methods interfaced with Newton-X NS through external calls. Those calls are responsible for adapting the input for the different electronic structure programs, calling them, reading their outputs, and returning them all in a structured, unified format. Newton-X will also be responsible for reading the orbital at different timesteps and calling the CIOverlap^80^ program to compute the overlap of subsequent timesteps used in eq 20. This interface is seamless for the user, who is not required to be familiar with Newton-X or surface hopping to use Legion. We do not imply that the program can only be used with Newton-X, but new direct interfaces have yet to be added as the need arises. This intermediary interface calls conventional electronic structure software, and the possible overhead of passing through the Newton-X call is negligible.

In recent years, semiempirical methods^99−106^ and machine learning potentials^107−109^ have been gaining popularity. In this case, the single-point calculation is so fast that small overheads can start to impact the performance of the simulation. For that case, we have created a direct interface to MLatom,^110,111^ an independent software that implements those faster methods. Since MLatom is provided as a Python API, we can call it from within the Legion interface. This can be significant for speeding up since calculating energies with a machine learning potential is so fast that importing the Python libraries at each time step can take a noticeable chunk of the time. Being called from the interface, MLatom is initiated only once, eliminating the overhead caused by reinitializing it multiple times.

A third direct interface is present for PySCF,^112^ a general-purpose electronic structure software with multiple methods that have been amply developed. It follows the philosophy of being a modular software that gives flexibility for developing new methods. The program is also provided as a Python API, which means it is also called from within its interface, similar to MLatom. The complete list of programs and methods interfaced with Legion at the time of writing is available in Table 1.

**Table 1:** Quantum Chemistry Programs and Electronic Structure Methods Interfaced with Legion^a^

Program	Methods^b^	Couplings
COLUMBUS^c^	MCSCF, MRCI	nacv, cioverlap, TDBA
TURBOMOLE^c^	CC2, ADC2, TDDFT	nacv, cioverlap, TDBA
ORCA^c^	TDDFT	cioverlap, TDBA
GAUSSIAN^c^	TDDFT	nacv, cioverlap, TDBA
OpenMolcas^c^	RASSCF, CASPT2, MC-PDFT	nacv, TDBA
MOPAC (Pisa)^c^	FOMO–CI	nacv, third-party overlap, TDBA
MNDO^c^	ODMx/MRCI	nacv, TDBA
OpenQP^c^	MRSF/TDDFT	TDBA
MLatom	AIQM1 ML potentials	TDBA
PySCF	TDDFT, ADC2, MCSCF	TDBA, nacv
Build-in codes	Analytical models	nacv, TDBA

a Dynamics can use nonadiabatic couplings coming from nonadiabatic coupling vectors (nacv), time-derivative couplings (cioverlap), or time-dependent Baeck-An approximation (TDBA).

b **MCSCF**: Multiconfigurational Self-Consistent Field; **MRCI**: Multireference Configuration Interaction; **CC2**: Second-Order Approximate Coupled Cluster; **ADC2**: Algebraic Diagrammatic Construction Second Order; **TDDFT**: Time-Dependent Density Functiona Theory; **RASSCF**: Restricted Active Space Self-Consistent Field; **CASPT2**: Complete Active Space Perturbation Theory Second Order; **MC-PDFT**: Multiconfiguration Pair-Density Functional Theory; **FOMO–CI**: Floating Occupation Molecular Orbitals-Configuration Interaction; **ODMx/MRCI**: Orthogonalization-Corrected Methods of Order x/MRCI; **AIQM1**: Artificial Intelligence–Quantum Mechanical Method 1; **ML potentials**: Machine Leaning potentials.

c Program interface mediated by Newton-X NS.

#### 3.2.3 Input and Output

**Figure 4:**
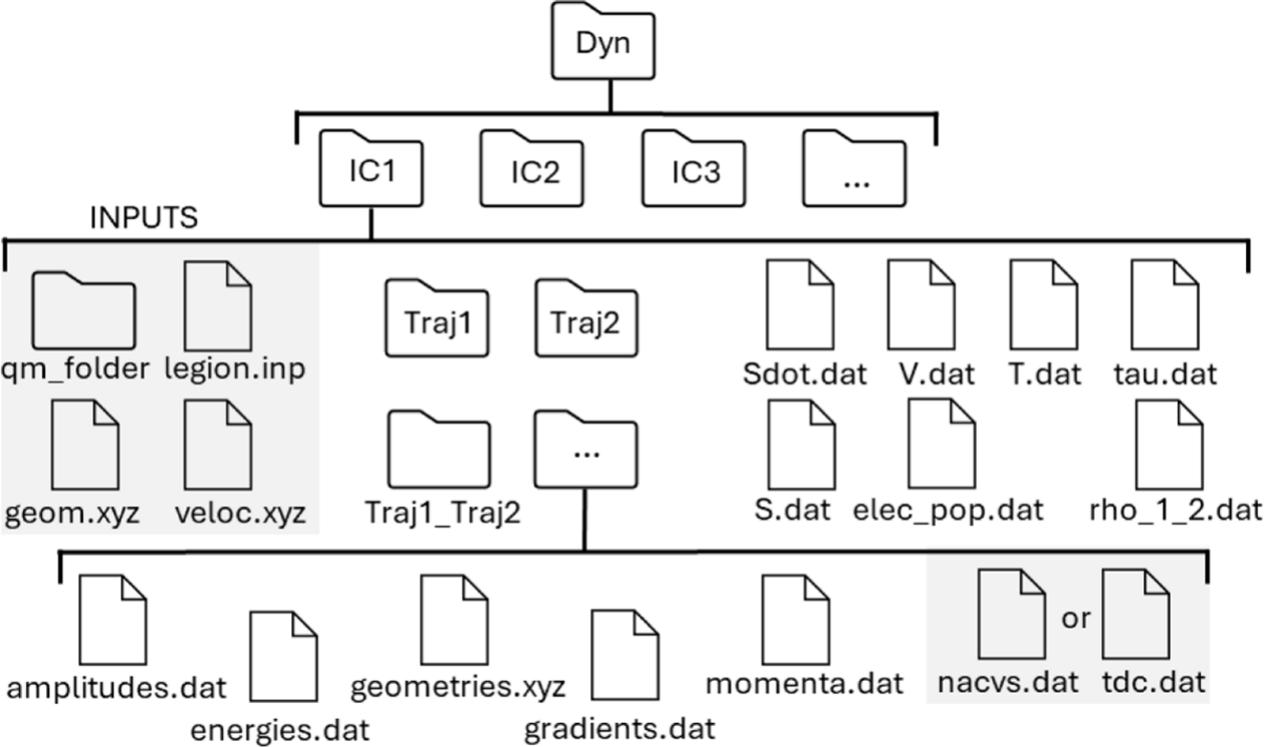
Folder structure of a simulation using Legion. Each *IC* folder is independent of the others and starts from a different initial condition. The inputs are marked, and the *qm_folder* contains the input for the single-point calculation. There is a folder for each trajectory (*TRAJ1*, *TRAJ2*, ...) and centroid (*TRAJ1_TRAJ2* between trajectories 1 and 2). The *nacvs.dat* is printed in case the electronic structure method provides the nonadiabatic coupling vector; otherwise, the time derivative coupling used is written in the *tdc.dat* file.

The input is composed of three parts, as shown in Figure 4:

- a single input file with the options to be used by Legion, defining multiple parameters of the dynamics. Most of them contain default values, except information such as the number of electronic states to consider and maximum simulation time;
- a pair of files containing the initial geometry and velocity of the first trajectory in *xyz* format;
- a folder containing the input to run the electronic structure calculation.

The output is composed of a folder for each trajectory containing its information: positions, momenta, gradients, energies, and coefficients. If the electronic structure methods compute the nonadiabatic coupling vectors, those will also be present. If the coupling is calculated with one of the auxiliary methods, then the time derivative coupling will be saved to a text file. The same information is stored in extra folders containing the centroid if the user decides to use the SPA approximation. Those trajectories are stored in a format compatible with Newton-X so that Ulamdyn^46^ can be used to analyze them.

Outside the trajectory folders, the matrices used to propagate the coefficient are also stored in specific files: *S.dat*, *Sdot.dat*, *V.dat*, *T.dat*, and *tau.dat*. The electronic population and the coherences of the wavepacket are also printed, together with the complete list of parameters and their respective values used in that simulation, including the default ones.

## 4 Application

### 4.1 Computational Details

**Figure 5:**
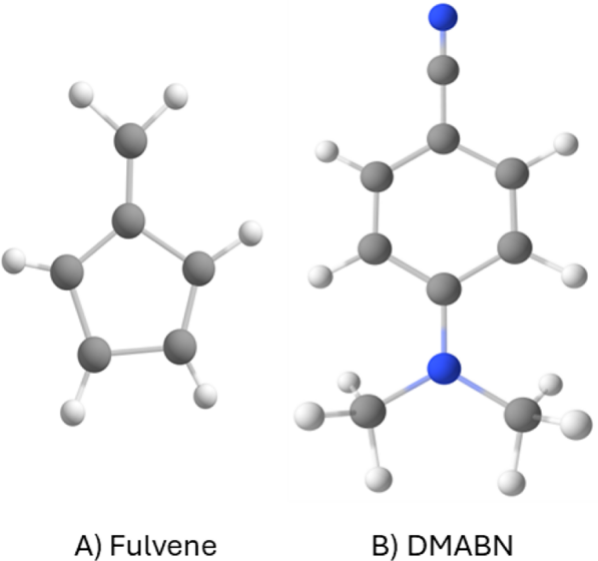
Molecular structures of Fulvene and DMABN.

To test and validate Legion, we performed AIMS dynamics of fulvene and DMABN [4-(N,N-dimethylamino)benzonitrile] (Figure 5) using multiple electronic structure methods implemented in various software with different types of nonadiabatic couplings. The simulations were performed interfacing with electronic structure computed with OpenMolcas 24.06^113−115^ for state-average complete active space self-consistent field (SA-CASSCF) and complete active space perturbation theory of second order (CASPT2). We used Columbus 7.2^116−118^ for CASSCF. Orca v5.0.4^119,120^ and Gaussian 16^121^ were adopted for the time-dependent density functional theory (TDDFT) calculations. Multiple spawning results reported in ref.^122^ were also done with Legion. The version of Legion used (v 1.0) is available at: https://gitlab.com/light-and-molecules/legion. It is interface with OpenMolcas was intermediated by Newton-X v.3.5.2.

For fulvene, we used two sets of initial conditions. One set contains the first 20 initial conditions from the set of dynamics in ref.,^75^ generated from a harmonic oscillator Wigner distribution. The other set contains 18 trajectories, also generated from a harmonic Wigner sampling, but the momenta were set to zero.^123^ A set of dynamics was run using SA-2-CASSCF(6,6) in Columbus and OpenMolcas. The active space consists of the three pairs of bonding π and antibonding π* orbitals. Dynamics with extended multistate (XMS)-CASPT2 used the same active space through the OpenMolcas interface. The 6-31g* was the basis set in all cases. The propagation started from the first excited state (S_1_) and continued until 45 fs with a full time step of 0.5 fs and a subtimestep in the coupling region of 0.1 fs. The population limit to allow spawning or trajectory elimination was 0.01, and the overlap threshold between trajectories to allow spawning was set to 0.6. The coupling criteria used was the time derivative coupling (**v·d***_IJ_*), with a threshold of 0.01 au^–1^. In most cases, the NAC was computed by the electronic structure calculation, and the momentum was corrected along the NAC direction at spawning time. Different sets of calculations were performed, varying the coupling threshold, type of coupling (NAC vs TDBA), the centroid method (SPA, BAT, BATE), and direction of momentum correction, either in the direction of the NAC or the momentum vector.

For DMABN, we used the 21 initial conditions presented in ref,^123^ also generated from a harmonic Wigner distribution. The sets of dynamics used LC-ωHPBE^124^/6-31g in Gaussian and ωB97X-D3^125^/def2-SV(P) in Orca, using the Tamm-Dancoff Approximation (TDA) and RIJCOSX^126^ to approximate Coulomb integrals. In both cases, the lowest three excited states were asked. The dynamics started from the second excited state (S_2_). They were propagated until 100 fs, with a full time step of 0.5 fs and a step of 0.1 fs in the coupling region. The population to kill and spawn is 0.1, the overlap threshold is 0.6. The coupling criteria is the TDC computed with TDBA with a threshold of 0.005 au^–1^. In all sets of dynamics the coupling was computed using TDBA, varying the centroid method (BAT, BATE).

### 4.2 Results for the Test Cases

When Tully proposed the FSSH algorithm, he validated the method using three one-dimensional analytical models that could also be solved numerically.^12^ Those models were amply adopted for testing algorithms, but they are very simplified representations of avoided crossing and coupling with reflection. In a newer publication, Ibele and Curchod proposed three molecules to be used as analogous to the Tully models.^123^ Of those molecules, we chose fulvene and DMABN (Figure 5) to validate Legion. Ethylene was not included in the tests since, due to its very small size, it gains a disproportionately large amount of kinetic energy per degree of freedom after initial excitation. This results in stability challenges for active-space-based electronic structure methods, which are essential for accurately describing the system, making ethylene less suitable as a reliable test case for dynamics.

Fulvene is widely used in nonadiabatic dynamics benchmarks,^27,66,75,86,127^ due to its ultrafast relaxation time and rigidity, which allow for stable complete active space (CAS) calculation throughout the propagation. Previous CASSCF calculations with multiple spawning^66,123^ and surface hopping^75,123^ have been characterized mainly by two deactivation channels: a stretch of the C = CH_2_ involving a sloped conical intersection and a twist of the same bond involving a peaked conical intersection. The intense population transfer previously observed for CASSCF starts at the first 10 fs of propagation, with a partial reflection back to the first excited state.

DMABN has also been added as an example of a TDDFT/AIMS calculation. TDDFT has previously been used to compute multiple spawning dynamics,^128,129^ but this is the first time the nonadiabatic coupling calculation has been completely circumvented. We show examples of interfaces with different electronic structure methods.

### 4.3 Fulvene

**Figure 6:**
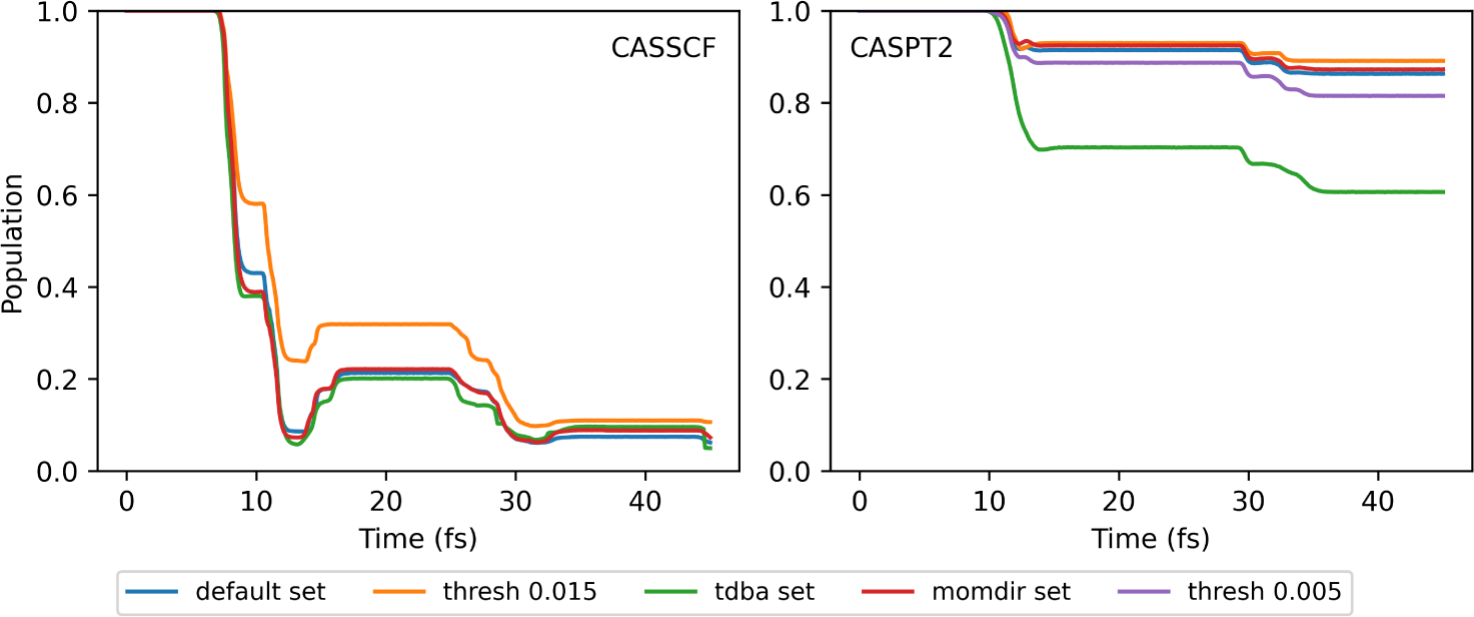
Population of the first excited state (S_1_) for the fulvene dynamics with SA-2-CASSCF(6,6)/6-31g* and XMS-CASPT2, computed with Legion/OpenMolcas from the initial conditions presented in ref,^123^ where the initial momentum is set to zero, *null KE* and using different sets of parameters.

We used two combinations of initial conditions and computed the S_1_ population profile using both SA-2-CASSCF(6,6) and XMS-CASPT2 for each set. The first collection of initial conditions, *null KE*, is extracted from ref.,^123^ and contains 18 pairs of geometries and velocities; the second collection contains the first 20 pairs of ref.^75^ For both electronic structure methods, we used different sets of parameters to test the convergence of the simulation. In the *default set*, we used the coupling threshold of 0.01 au^–1^; the coupling used was the nonadiabatic coupling vector computed from the electronic structure calculation, and the momentum was corrected in the direction of the NAC at a spawn. The centroid was calculated using the BAT approximation. In the *thresh 0.015*, the coupling threshold was modified to 0.015 au^–1^. In the *tdba set*, the coupling was computed with the TDBA approximation, and the momentum corrected in the direction of the momentum. In the *momdir set*, the nonadiabatic coupling was calculated, but the momentum was corrected in the direction of the momentum. Finally, in the *thresh 0.005*, only the coupling threshold was modified to 0.005 au^–1^. This last set was used only in the CASPT2 dynamic. The plot of the S_1_ population for all sets with both CASSCF and CASPT2 is shown in Figure 6.

For CASSCF, almost all sets return the same population profile except for the high coupling threshold. In particular, we call attention to the *tdba set*, which is practically indistinguishable from the remaining sets. The profile matches both ref^123^ and ref.,^66^ which computed AIMS/CASSCF dynamics for fulvene using FMS90/Molpro^63^ and PySpawn/OpenMolcas,^66^ respectively, and used the same initial conditions.

For CASPT2, the population transferred to the ground state in the same time window is completely different. While in CASSCF, the S_1_ population at 45 fs is around 6% for most sets, for CASPT2, the same population is close to 85% (Table 2). The general trend of the populations also agrees with the CASPT2 dynamics of the interface of PySpawn/OpenMolcas. This striking difference has been previously reported by Ibele et al.^66^ We can notice that the lower threshold of 0.005 leads to slightly more population transfer, and a threshold of 0.015 (used by Ibele et al.) leads to an almost unnoticeable loss of transfer. The TDBA approximation overestimates the amount of population transferred to the S_0_.

**Table 2:** Population of the First Excited State (S_1_) at Time 45 fs for the Fulvene Dynamics with SA-2-CASSCF(6,6)/6-31g* and XMS-CASPT2, Computed with Legion/OpenMolcas from the Initial Conditions Presented in Ref.^123^ where the Initial Momentum Is Set to Zero, *Null KE* and Using Different Sets of Parameters

	CASSCF	CASPT2
*default set*	0.062	0.863
*thresh 0.015*	0.106	0.891
*tdba set*	0.050	0.606
*momdir set*	0.073	0.873
*thresh 0.005*	--	0.815

**Figure 7:**
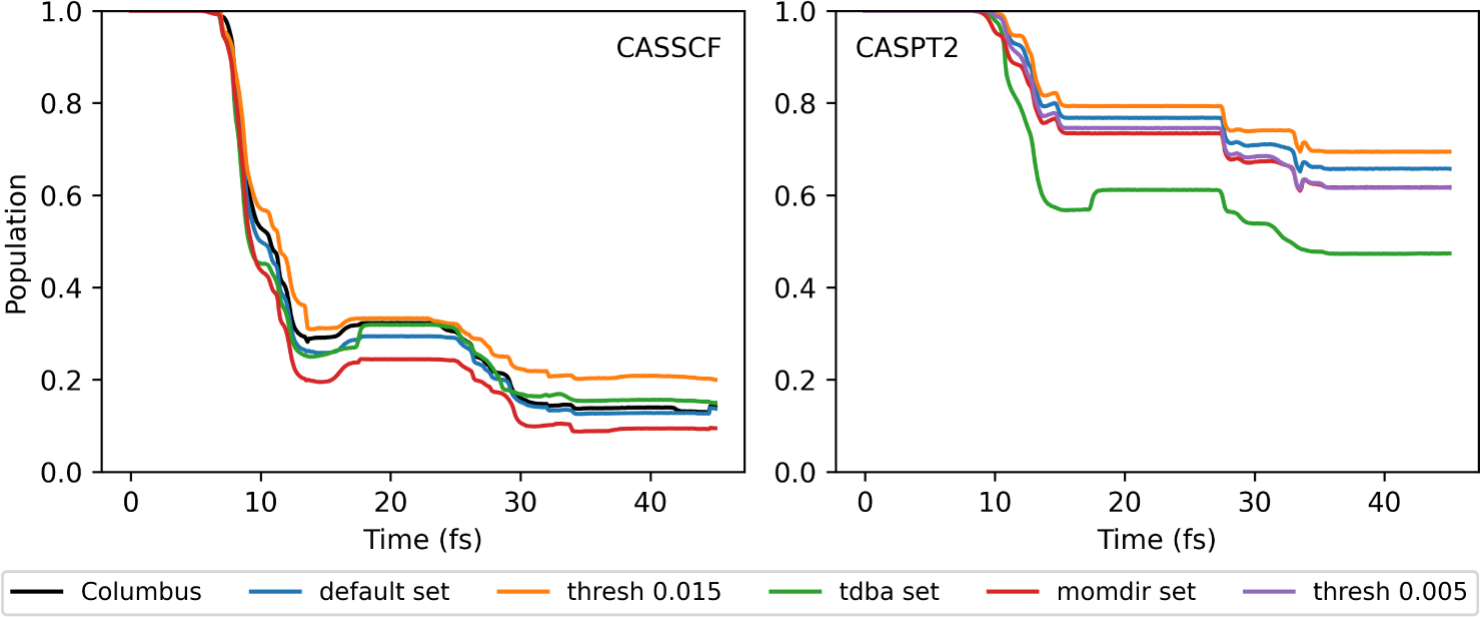
Population of the first excited state (S_1_) for the fulvene dynamics with SA-2-CASSCF(6,6)/6-31g* and XMS-CASPT2, computed with Legion/OpenMolcas and Legion/Columbus from the initial conditions presented in ref. (75) where the initial momentum is not altered, *full KE* and using different sets of parameters.

The intensity of the difference between CASSCF and CASPT2 dynamics is surprising. ref.^66^ explained this difference using static calculations. It showed that the reaction path to the sloped conical intersection has a minimum in S_1_ for CASPT2 that is not present, or not as intensely, in the PES with CASSCF. Although this could explain the difference, we wanted to test whether other aspects of the dynamics could be partially responsible. To evaluate this, we performed the same sets of dynamics with both CASSCF and CASPT2 using the first 20 trajectories previously used in surface hopping.^75^ Both initial conditions were generated from a harmonic Wigner distribution, but the first collection of initial conditions (Figure 6) had the initial velocity set to zero. This choice was made explicitly to favor the decay through the sloped conical intersection and probe the reflection mechanism in fulvene,^123^ analogous to Tully model 3.^12^ The second collection of initial conditions uses the velocity generated by the sampling without modification (Figure 7).

The CASSCF dynamics already show a difference when compared to the *null KE* initial conditions, both in the actual population profile and in the fact that the different sets of dynamics cause a more noticeable impact. In this case, the higher threshold does not affect the population as much, and the TDBA approximation once again agrees very much with the dynamics using NAC. In this case, the momentum direction at spawning time leads to more population being transferred to the ground state, although the difference is small, 4% (Table 3). We performed an extra set of dynamics using the default parameters but using CASSCF implemented in Columbus. This program was used in previous publications in the FSSH dynamics of fulvene and agrees with the Legion/OpenMolcas interface simulations.

**Table 3:** Population of the First Excited State (S_1_) at Time 45 fs for the Fulvene Dynamics with SA-2-CASSCF(6,6)/6-31g* and XMS-CASPT2, Computed with Legion/OpenMolcas and Legion/Columbus from the Initial Conditions Presented in Ref. (75) where the Initial Momentum Is Set to Zero, *Full KE* and Using Different Sets of Parameters

	CASSCF	CASPT2
*default set*	0.137	0.657
*thresh 0.015*	0.200	0.694
*tdba set*	0.150	0.474
*momdir set*	0.095	0.617
*thresh 0.005*	--	0.616
*Columbus*	0.141	

For CASPT2, there is a noticeably less population in S_1_ for all sets of parameters. Both the lower coupling threshold of 0.005 au^–1^ and the direction of momentum correction increase the amount of population being transferred to S_0_. Similar to the previous CASPT2, the TDBA also overestimates the amount of population transfer.

**Figure 8:**
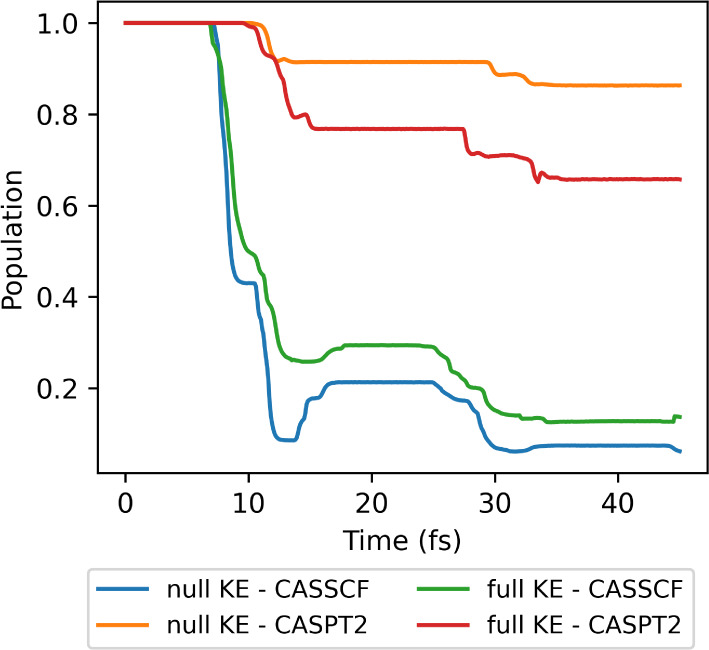
Population of the first excited state (S_1_) for the fulvene dynamics with SA-2-CASSCF(6,6)/6-31g* and XMS-CASPT2, computed with Legion/OpenMolcas from the initial conditions presented in ref.^123^ and ref.,^75^ the *null KE* and *full KE* initial conditions and for the default set of parameters.

A comparison of the default parameters for the different initial conditions with CASSCF and CASPT2 is presented in Figure 8. We can see how the initial kinetic energy can be used to favor one decay pathway over the other. In the *null KE*, which favors the sloped conical intersection, the reflection of the CASSCF dynamics is strongly noticeable. At the same time, the CASPT2 dynamics, which has an S_1_ minimum before the sloped intersection, has a significantly delayed decay time. In the *full KE*, on the other hand, the peaked conical intersection is expected to contribute to the relaxation mechanism. This is corroborated by the less pronounced reflection observed in the CASSCF calculation and the stronger population transfer in the CASPT2 dynamics, enabled by the peaked intersection that does not have an S_1_ minimum.^66^

**Figure 9:**
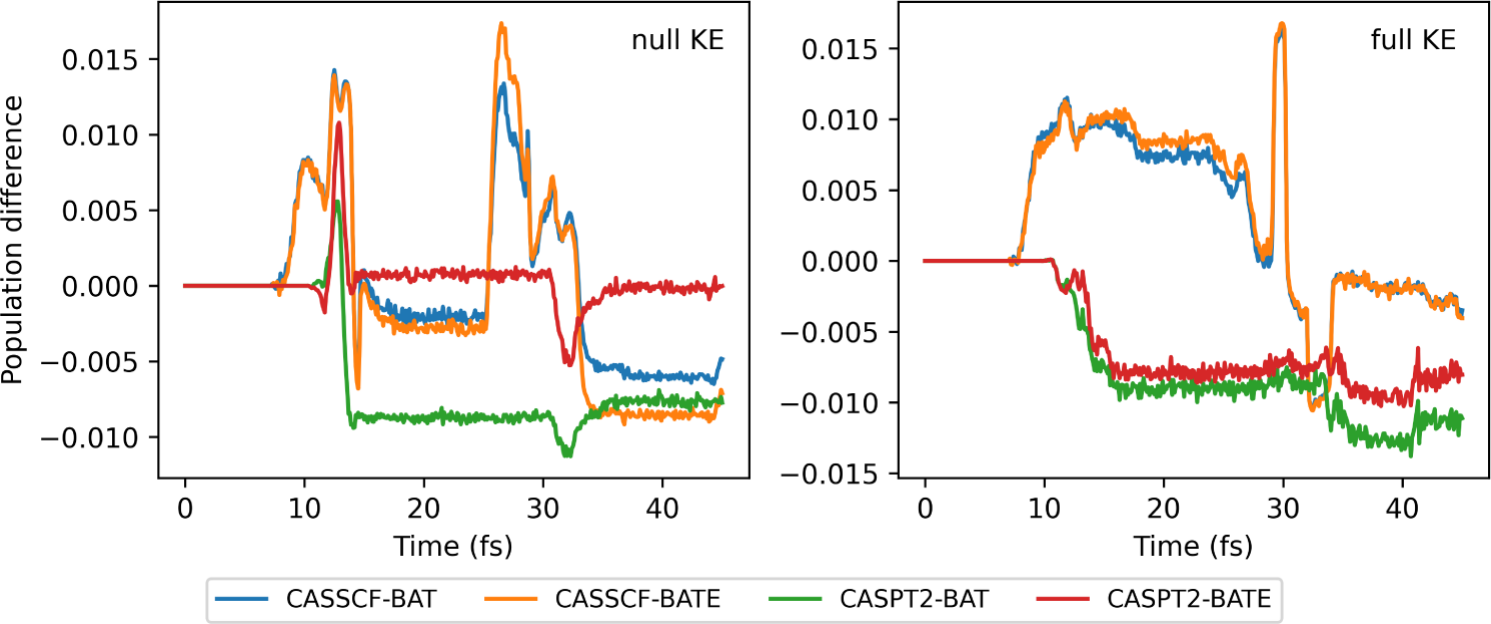
Difference between the S_1_ population, computed as *SPA—BAT(E)* for the *null KE* and *full KE* collections of initial conditions, using default parameters, for fulvene dynamics using CASSCF and CASPT2 electronic structures.

Beyond the discussion of the molecular systems, it is worth focusing on the BAT approximation, which has been used in all calculations presented up until now. In Section 2.4, we presented the modified BAT approximation, BATE. Still, we chose to use the original approximation for the calculations presented here since this is the one established in the literature. We can compare the S_1_ population of fulvene when computed with the SPA approximation, BAT, and BATE, as shown in Figure 9. In all cases, the difference in population between SPA and the other methods is less than 1.5%. This is well within a tolerable error for the population, considering the speed-up provided by not needing to compute the centroid values. Particularly when noticing that this error is below 1% most of the time.

When comparing the conventional BAT approximation and the newly suggested BATE, one can see that they can reproduce the SPA almost numerically. With both initial conditions, the difference between BAT and BATE is minimal for both CASSCF dynamics. Still, BAT is closer to the centroid dynamics. On the other hand, for the CASPT2 dynamics, BATE can approach the SPA population better. Particularly for the *null KE*, which has almost no population transfer, BATE reproduces almost exactly the SPA calculation. The improvements of BATE over BAT are marginal, but it shows that although the BAT approximation is already quite good, there is room for improvement if one considers the correct shape of the nonadiabatic coupling curve.

**Figure 10:**
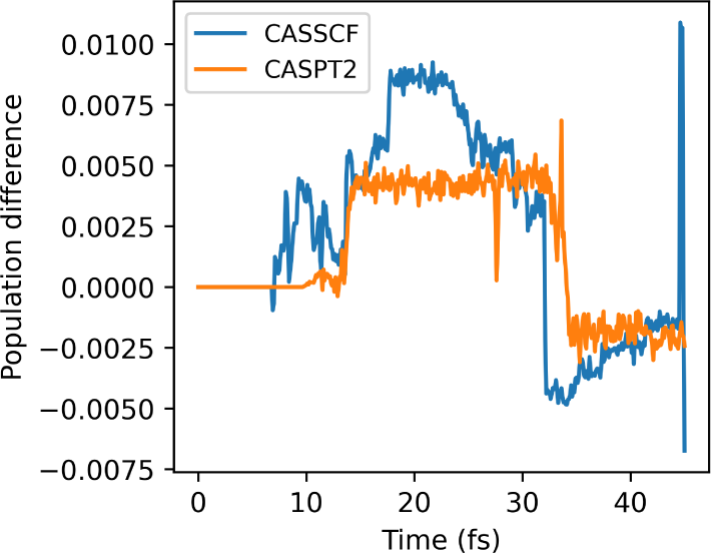
Population difference of S_1_ between timesteps of 0.5 fs with 5 substeps vs timesteps of 0.1 fs without substeps of the fulvene dynamics. Computed for the *null KE* initial conditions with CASSCF and CASPT2 electronic structures.

Another method used to speed up the calculation, the adaptive step, can be evaluated by comparing the population at different time step sizes. Figure 10 has the difference between the reference calculation, where all steps taken are 0.1 fs with no data interpolation between steps, and the adaptive scheme, where the classical trajectory moves with a time step of 0.5 fs outside the coupling region and 0.1 fs within it. The missing data is being interpolated every 0.1 fs to propagate the coefficients. This scheme also has a difference below 1% with the reference, showing a good balance between error and efficiency, and can be modified with different step sizes and number of substeps.

**Figure 11:**
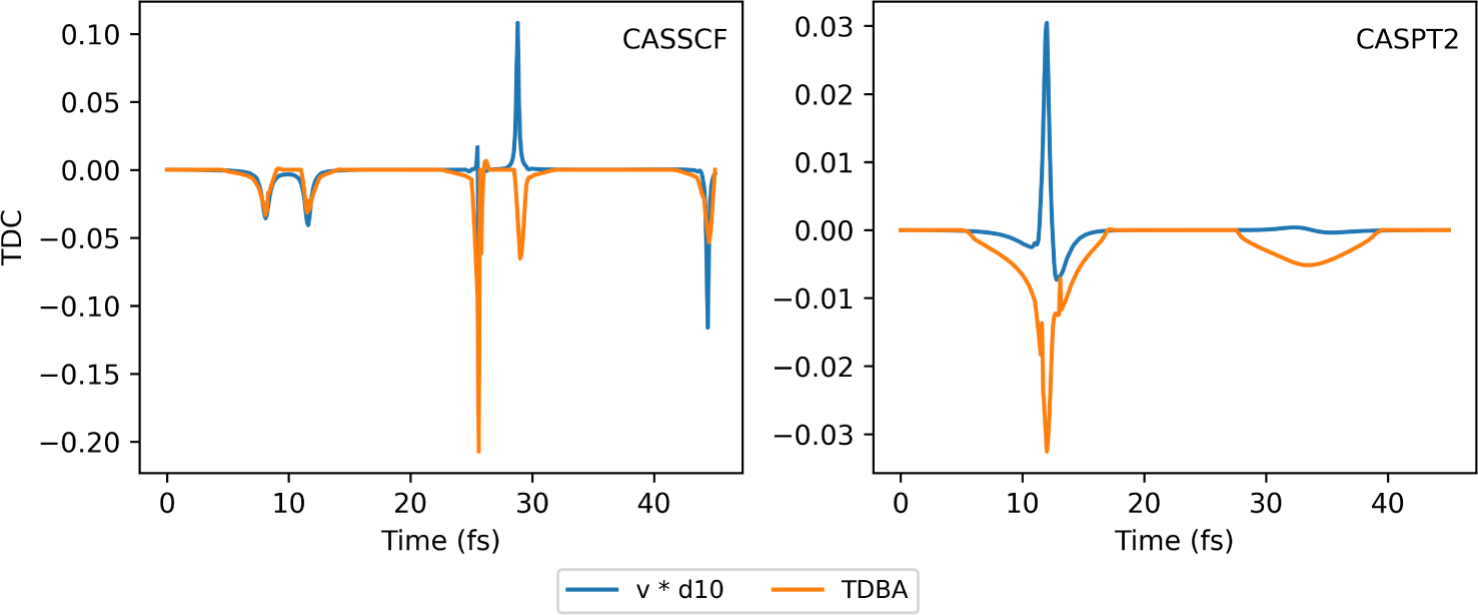
Time derivative coupling (TDC) between S_0_ and S_1_ computed with TDBA approximation and the dot product velocity (**v**) times nonadiabatic coupling (**d***_10_*) for a single trajectory of fulvene from the *null KE* initial conditions computed with CASSCF and CASPT2 electronic structures.

Finally, we can look closer at the TDBA approximation to understand why it fails for CASPT2. Figure 11 contains the time derivative coupling for the same trajectory from the null KE initial conditions computed from the nonadiabatic coupling, with the formula.
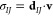
27And computed using TDBA. In CASSCF, where the coupling is high, TDBA can follow the general behavior of the time derivative coupling. It peaks at the same time, and the difference in sign does not seem to cause much of a problem with the coefficient integration. In CASPT2, on the other hand, the coupling is considerably smaller. In this case, TDBA seems to overestimate the amount of coupling, consequently leading to more population transfer. This is taken as a representative case; the same is observed in the *full KE* sets of initial conditions.

### 4.4 DMABN

The interest in DMABN is because the PES of the first and second excited states meet multiple times along the dynamics, which causes S_1_ and S_2_ to keep exchanging populations. This sequence of avoided crossings is a good representation of the Tully model 2.^123^

**Figure 12:**
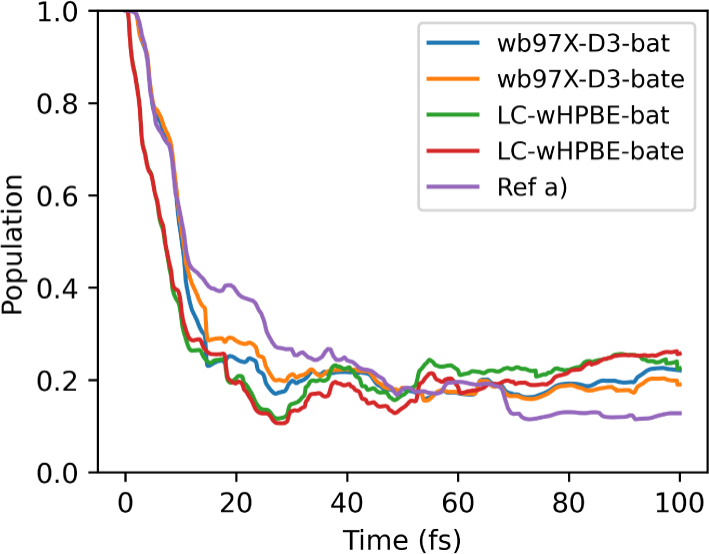
S_2_ population for DMABN propagated with ωB97X-D3/def2-SV(P) in Legion/ORCA and LC-ωHPBE/6-31g in Legion/Gaussian. ref a) is ref^123^ LC-PBE/6-31g computing NAC.

Figure 12 shows the population of the second excited state computed for DMABN using different TDDFT functionals (ωB97X-D3 and LC-ωHPBE) implemented on different electronic structure software. All calculations use the same set of parameters except for the BAT and BATE approximations. The reference for comparison is available in the Supporting Information of ref^123^ and was propagated with LC-ωPBE implemented in TeraChem,^130−133^ using the same coupling threshold as the calculations presented here, 0.005 au^–1^. TDBA causes the population to transfer more intensely within the first 20 fs of propagation. Afterward, the population oscillates around 0.2 in the S_2_ state. Despite the variation, it agrees qualitatively with the reference.

**Figure 13:**
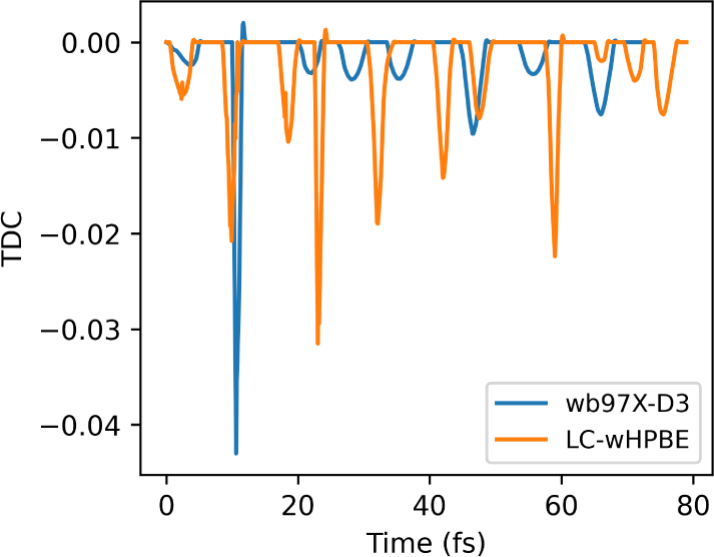
Time derivative coupling between states S_1_ and S_2_ for a single trajectory of DMABN computed with TDBA. Dynamics propagated with ωB97X-D3/def2-SV(P) in Legion/ORCA and LC-ωHPBE/6-31g in Legion/Gaussian.

A closer inspection of the time derivative coupling (Figure 13) indicates the repetitive interactions between S_1_ and S_2_, denoted by the peaks. An inspection of the TDC of fulvene shows the correlation between the high coupling in the CASSCF dynamics (Figure 11) and a good approximation with TDBA (Figure 7), while the dynamics with a small coupling, CASPT2 (Figure 11), have the TDC being overestimated (Figure 7). DMABN also shows a higher population transfer of 20% around 20 fs (Figure 12), with the same intensity of the TDC as seen for the fulvene/CASPT2 dynamics (Figure 13). This shows that while TDBA can still return quantitively correct results in some cases, this is not always reliable, and nonadiabatic coupling calculation is still required. This conclusion conflicts with Shu and Truhlar’s conjecture^16^ that curvature-based couplings could safely replace full coupling vectors. Still, even in those suboptimal situations, TDBA seems to return at least a qualitatively good description of the dynamics. The trend observed here of higher TDC leading to better results could lead to tools to assess the quality of the TDBA in a given system without recurring comparison with a calculation that computes the nonadiabatic coupling. Future investigations are still necessary to establish general rules for this evaluation if this trend holds for other molecular systems, in which situation it holds, and if the values observed here are universal or system-specific.

## 5 Conclusion

We presented Legion, a computer program intended to facilitate the development of nonadiabatic classical-trajectory-guided quantum wavepacket methods. The program is part of the Newton-X platform, inheriting the interfaces to electronic structure programs already available for Newton-X NS. Legion is mostly written in Python3, a flexible language that allows for modular code, where tasks run independently. This makes it easy to reuse code in different sections and switch between modules to use different dynamics methods and approximations. Although the computationally expensive part of the dynamics is usually the electronic structure calculation, Legion uses modules written in Fortran for the mathematical operations, such as building the Hamiltonian for the coefficient propagation. Beyond the Fortran module, Legion also employs strategies like adaptive time step for classical propagation, parallel trajectory propagation, and avoiding computation of the centroid.

We have detailed the code’s structure and the implementation of a conventional AIMS method. We have also presented a modification for the BAT approximation, which, although only marginally improved over the original, shows that there is still room to improve in BAT. Dynamics in Legion can be based on nonadiabatic coupling vectors and time-derivative couplings obtained from wave function overlap. Moreover, we took a new curvature-based coupling approximation developed for surface hopping calculations, TDBA, and implemented it for the first time in the context of multiple spawning. TDBA allows dynamics propagation with any electronic structure method that can deliver excited state energies and their gradients. Legion also contains Gaussian width parameters for the entire periodic table. Such an ensemble of software interface, electronic structure methods, coupling algorithms, algorithmic approximations, and extended Gaussian parametrization makes Legion an extremely flexible platform.

We validated Legion with a series of AIMS simulations of fulvene and DMABN. We showed the dynamics for DMABN, completely avoiding the computation of nonadiabatic coupling vectors. We also performed a series of dynamics with fulvene, varying some of the multiple spawning parameters and complementing the discussion presented in the literature. We saw how the choice of initial condition is related to the different population profiles observed from dynamics with CASSCF and CASPT2 since the initial condition can increase the relevance of specific relaxation pathways.

While we showed that, in some cases, the TDBA approximation works as well as NAC dynamics, we also saw examples where it overestimates the population transfer. However, we lack tools to evaluate whether TDBA will be a good approximation for a given system. Still, even in cases where the approximation does not agree numerically with the reference, the dynamics seem to be able to recover the qualitative behavior of the system. The computation of the time derivative coupling using overlap is becoming routine in both surface hopping and multiple spawning methods, and this is currently being implemented in Legion.

This first version of Legion contains the structure necessary to propagate dynamics. Still, we are planning the following steps to extend the tools available in the package. We will focus on the train of trajectories^33,134^ and a recently proposed variational propagator for a linearly dependent moving basis.^135^ The former is a computationally inexpensive way of increasing the nuclear basis by adding different points in time of the same classical trajectory to the basis. It allows trajectory communication in a larger range of the phase space. The latter is an alternative way of computing the trajectory coefficients that deals with a known problem of frozen Gaussian propagation methods: energy conservation.^66,136,137^

## Data Availability

The code is available as open source at: https://gitlab.com/light-and-molecules/legion. The data of the trajectories can be found at: 10.5281/zenodo.13784439.

## Supporting Information Available

The derivation of the AIMS equations of motion (PDF)

## Footnotes

a We refer to classical wavepackets in the sense of trajectories simultaneously spanning a common region in the configuration space but uncorrelated from each other, as obtained from a classical limit of the time-dependent Schrödinger equation.^17^ We can rigorously define the classical wavepacket density on state *J* for an ensemble of *N*_*traj*_ trajectories as , where δ_*x*_ (*A*) is the Dirac measure, which takes the value 1 for *x* = *A* and 0 for *x* ≠ *A*.
