# Supplementary material for: Legion: A Platform for Gaussian Wavepacket Nonadiabatic Dynamics
Source: J Chem Theory Comput. 2025 Mar 3;21(5):2189–205. doi: 10.1021/acs.jctc.4c01697 (PMC11948330; doi:10.1021/acs.jctc.4c01697)
Supplement: Supplementary file 1 — ct4c01697_si_001.pdf [file ct4c01697_si_001.pdf]

Legion: A Platform for Gaussian Wavepacket Nonadiabatic Dynamics

Rafael S. Mattos,[1] Saikat Mukherjee,[1,2] Mario Barbatti[1,3]*

[1] *Aix Marseille University, CNRS, ICR, Marseille, France*
[2] *Faculty of Chemistry, Nicolaus Copernicus University in Torun, Poland*
[3] *Institut Universitaire de France, 75231 Paris, France*

* *Corresponding author: [mattos.rsouza@gmail.com, mario.barbatti@univ-amu.fr]*

Supplementary Material

Table of Contents

Legion: A Platform for Gaussian Wavepacket Nonadiabatic Dynamics 1
S1. Equations-of-motion of the nuclear coefficients 2
References 5

S1. Equations-of-motion of the nuclear coefficients

Using the Born-Huang expansion of the total wavefunction is

$$\Psi(\mathbf{r},\mathbf{R},t)=\sum_{J}^{\infty}\Omega_J(\mathbf{R},t)\phi_J(\mathbf{r};\mathbf{R}) \tag{S1}$$

where the nuclear wavepacket of each electronic state can be represented as

$$\begin{aligned}\Omega_J(\mathbf{R},t)&=\sum_{j}^{N_J}C_j^J(t)\chi_j^J(\mathbf{R})\\ \chi_j^J(\mathbf{R})&=\prod_{\rho=1}^{3N}\left(\frac{2\omega_\rho}{\pi}\right)^{1/4} exp[-\omega_\rho(R_\rho-\bar{R}_{j\rho}^J(t))^2+i\bar{P}_{j\rho}^J(t)(R_\rho-\bar{R}_{j\rho}^J(t))+i\gamma_j(t)]\end{aligned} \tag{S2}$$

and applying it in the usual time-dependent Schrödinger equation

$$i\frac{\partial\Psi(\mathbf{r},\mathbf{R},t))}{\partial t}=\hat{H}(\mathbf{r},\mathbf{R})\Psi(\mathbf{r},\mathbf{R},t) \tag{S3}$$

one can start the process of finding the equation for the time derivative of the coefficients $C_j{}^J(t)$. In these equations, $\mathbf{r}$ and $\mathbf{R}$ represent the electronic and nuclear coordinates, respectively. Initially, the substitution leads to

$$i\frac{\partial}{\partial t}\sum_{J}^{\infty}\sum_{j}^{N_J}C_j^J\chi_j^J\phi_J=\hat{H}\sum_{J}^{\infty}\sum_{j}^{N_J}C_j^J\chi_j^J\phi_J \tag{S4}$$

The chain rule can open the right side of the equation

$$\frac{\partial}{\partial t}\sum_{J}^{\infty}\sum_{j}^{N_J}C_j^J\chi_j^J\phi_J=\sum_{J}^{\infty}\sum_{j}^{N_J}\dot{C}_j^J\chi_j^J\phi_J+C_j^J\dot{\chi}_j^J\phi_J \tag{S5}$$

and then projected over the basis of the wavefunction

$$\begin{aligned}\left\langle\chi_i^I\phi_I\right|\frac{\partial}{\partial t}\left|\Psi\right\rangle_{\mathbf{r},\mathbf{R}}&=\sum_{J}^{\infty}\sum_{j}^{N_J}\left\langle\chi_i^I\phi_I\right|\dot{C}_j^J\left|\chi_j^J\phi_J\right\rangle_{\mathbf{r},\mathbf{R}}+C_j^J\left\langle\chi_i^I\phi_I\right|\dot{\chi}_j^J\phi_J\right\rangle_{\mathbf{r},\mathbf{R}}\\&=\sum_{J}^{\infty}\sum_{j}^{N_J}\dot{C}_j^JS_{ij}\delta_{IJ}+C_j^J\dot{S}_{ij}\delta_{IJ}\end{aligned} \tag{S6}$$

The coefficients do not depend on the position coordinates, so they do not participate in the integration, which is performed over the variables indicated in the bracket indexes. The product of the basis function leads to the overlap matrix elements since the nuclear wave packets are not orthogonal in general, times the electronic overlap matrix elements, which are orthonormal by construction. It also leads to the time derivative of the overlap matrix elements $\dot{\mathbf{S}}_{ij}$.

The right side of Eq. (S4) can go through the same projection over the basis functions, leading to one term of the kinetic operator and another term of the electronic operator

$$\begin{aligned}\left\langle \chi_i^I \phi_I \middle| \hat{H} \middle| \Psi \right\rangle_{\mathbf{r},\mathbf{R}} &= \left\langle \chi_i^I \phi_I \middle| (\hat{T}_n + \hat{H}_{el}) \middle| \sum_J^{\infty} \sum_j^{N_J} C_j^J \chi_j^J \phi_J \right\rangle_{\mathbf{r},\mathbf{R}} \\ &= \sum_J^{\infty} \sum_j^{N_J} C_j^J \left\langle \chi_i^I \phi_I \middle| \hat{T}_n \middle| \chi_j^J \phi_J \right\rangle_{\mathbf{r},\mathbf{R}} + C_j^J \left\langle \chi_i^I \phi_I \middle| \hat{H}_{el} \middle| \chi_j^J \phi_J \right\rangle_{\mathbf{r},\mathbf{R}}\end{aligned} \quad \text{(S7)}$$

The kinetic operator can be opened, leading to the appearance of the nonadiabatic elements:

$$\begin{aligned}\left\langle \chi_i^I \phi_I \middle| \hat{T}_n \middle| \chi_j^J \phi_J \right\rangle_{\mathbf{r},\mathbf{R}} &= -\frac{1}{2M} \langle \chi_i^I \phi_I | \nabla_{\mathbf{R}}^2 | \chi_j^J \phi_J \rangle_{\mathbf{r},\mathbf{R}} \\ &= \langle \chi_i^I | \hat{T}_n | \chi_j^J \rangle_{\mathbf{r},\mathbf{R}} \delta_{IJ} - \frac{1}{M} \langle \chi_i^I | \mathbf{d}_{IJ} \cdot \nabla_{\mathbf{R}} | \chi_j^J \rangle_{\mathbf{R}} - \frac{1}{2M} \langle \chi_i^I | D_{IJ} | \chi_j^J \rangle_{\mathbf{R}}\end{aligned} \quad \text{(S8)}$$

The first term of the expansion is simply the kinetic energy operator acting solely on the nuclear wave packet. The second term is a vector containing the first derivative of the electronic functions, referred to as the derivative coupling $\mathbf{d}_{IJ} = \langle \phi_I | \nabla_{\mathbf{R}} | \phi_J \rangle_{\mathbf{r}}$.[1] The third term is the scalar kinetic coupling, which is usually neglected $D_{IJ} = \langle \phi_I | \nabla_{\mathbf{R}}^2 \phi_J \rangle_{\mathbf{r}} \approx 0$.[1]

The electronic Hamiltonian does not depend on the nuclear coordinates and will only affect the electronic functions. Those are their eigenfunctions, so it is clear that

$$\left\langle \chi_i^I \phi_I \middle| \hat{H}_{el} \middle| \chi_j^J \phi_J \right\rangle_{r,R} = \epsilon_J S_{ij} \delta_{IJ} \quad \text{(S9)}$$

where ϵ_J is the electronic eigenvalue. Applying those relations back into the TDSE, one can obtain the equation of motion for the coefficients in matrix notation

$$\dot{\mathbf{C}} = -i\mathbf{S}^{-1}(\mathbf{T} - \boldsymbol{\tau} + \mathbf{V} - i\dot{\mathbf{S}}) \cdot \mathbf{C} \quad \text{(S10)}$$

where

$$\begin{aligned} S_{ij} &= \langle \chi_i^I | \chi_j^J \rangle_{\mathbf{R}} \delta_{IJ} \\ \dot{S}_{ij} &= \langle \chi_i^I | \dot{\chi}_j^J \rangle_{\mathbf{R}} \delta_{IJ} \\ T_{ij} &= \langle \chi_i^I | \hat{T}_n | \chi_j^J \rangle_{\mathbf{r},\mathbf{R}} \delta_{IJ} \\ V_{ij} &= \left\langle \chi_i^I \phi_I \middle| \hat{H}_{el} \middle| \chi_j^J \phi_J \right\rangle_{\mathbf{r},\mathbf{R}} \\ \tau_{ij} &= \frac{1}{M} \langle \chi_i^I | \mathbf{d}_{IJ} \cdot \nabla_{\mathbf{R}} | \chi_j^J \rangle_{\mathbf{R}} \end{aligned} \quad \text{(S11)}$$

The analytical formula for the matrix elements is not derived here, but their final form is presented under the approximation of constant amplitudes ω_ρ. For the overlap matrix, we have

$$S_{ij} = \prod_{\rho}^{3N} \exp\left(\frac{-\omega_\rho (\bar{R}_{i\rho}^{I} - \bar{R}_{j\rho}^{J})^2}{2} - \frac{(\bar{P}_{i\rho}^{I} - \bar{P}_{j\rho}^{J})^2}{8\omega_\rho} \right) \times$$
$$\exp\left(i \left[\frac{\bar{R}_{i\rho}^{I} + \bar{R}_{j\rho}^{J}}{2} \left(\bar{P}_{j\rho}^{J} - \bar{P}_{i\rho}^{I} \right) + \bar{R}_{i\rho}^{I} \bar{P}_{i\rho}^{I} - \bar{R}_{j\rho}^{J} \bar{P}_{j\rho}^{J} \right] \right) \times \exp\left(i \left(\gamma_j - \gamma_i \right) \right) \delta_{IJ} \quad \text{(S12)}$$

The atom width ω_ρ is defined at the beginning of the propagation and is kept the same, the centroid position for each Gaussian $\bar{\mathbf{R}}$ and the centroid momenta $\bar{\mathbf{P}}$ are obtained from the classical trajectories. In the subsequent matrix elements, the overlap can be reused, and only a multiplicative factor for each matrix element must be computed. For the time derivative overlap, we have

$$\dot{S}_{ij} = \sum_{\rho}^{3N} \left[\left(\omega_\rho \left(\bar{R}_{i\rho}^{I} - \bar{R}_{j\rho}^{J} \right) - i \frac{\bar{P}_{i\rho}^{I} + \bar{P}_{j\rho}^{J}}{2} \right) \bar{v}_{j\rho} + \left(\frac{\bar{P}_{i\rho}^{I} - \bar{P}_{j\rho}^{J}}{4\omega_\rho} + i \frac{\bar{R}_{i\rho}^{I} - \bar{R}_{j\rho}^{J}}{2} \right) \bar{f}_{j\rho} + i\dot{\gamma}_j \right] S_{ij} \quad \text{(S13)}$$

As it involves the time derivative of the Gaussian j, it will also take as input the classical velocity $\bar{\mathbf{v}}$, force $\bar{\mathbf{f}}$ for this trajectory and the time derivative of the phase $\dot{\gamma}$, which is given in the main text. For the kinetic energy matrix, we have

$$T_{ij} = \sum_{\rho}^{3N} \frac{1}{2M_\rho} \left[\left(\frac{\left(\bar{P}_{i\rho}^{I} + \bar{P}_{j\rho}^{J} \right)^2}{4} + \omega_\rho - \omega_\rho^2 \left(\bar{R}_{j\rho}^{J} - \bar{R}_{i\rho}^{I} \right)^2 \right) - i\omega_\rho \left(\bar{R}_{j\rho}^{J} - \bar{R}_{i\rho}^{I} \right) \left(\bar{P}_{i\rho}^{I} + \bar{P}_{j\rho}^{J} \right) \right] S_{ij} \quad \text{(S14)}$$

In the following matrices, the value of the potential energy and nonadiabatic coupling at the centroid position for each pair of Gaussians is required to compute the matrix elements.[2] Instead of the centroid value, the equations presented here use the BAT approximation,[3] discussed in the main text. The potential energy is

$$V_{ij} \approx \left[E_I(\mathbf{R}_i) + E_J(\mathbf{R}_j) + \sum_{\rho}^{3N} \left(\frac{\partial E_I(\bar{\mathbf{R}}_i^I)}{\partial R_\rho} + \frac{\partial E_J(\bar{\mathbf{R}}_j^J)}{\partial R_\rho} \right) \left(\frac{\bar{R}_{i\rho}^{I} + \bar{R}_{j\rho}^{J}}{2} + i \frac{\bar{P}_{j\rho}^{J} - \bar{P}_{i\rho}^{I}}{4\omega_\rho} \right) \right.$$
$$\left. - \bar{R}_{i\rho}^{I} \frac{\partial E_I(\bar{\mathbf{R}}_i^I)}{\partial R_\rho} - \bar{R}_{j\rho}^{J} \frac{\partial E_J(\bar{\mathbf{R}}_j^J)}{\partial R_\rho} \right] \frac{S_{ij}}{2}, \quad \text{(S15)}$$

taking only the real value of the element V_{ij}. And finally, the nonadiabatic coupling terms, also using the BAT approximation

$$\tau_{ij} = \sum_{\rho}^{3N} \left[\frac{1}{2M_\rho} \left(d_{i\rho}^{IJ} + d_{j\rho}^{IJ} \right) \left(\omega_\rho \left(\bar{R}_{j\rho}^{J} - \bar{R}_{i\rho}^{I} \right) + i \frac{\bar{P}_{i\rho}^{I} + \bar{P}_{j\rho}^{J}}{2} \right) \right] S_{ij} \quad \text{(S16)}$$

In this case, the overlap matrix element doesn't contain the δ_{IJ} term since it uses the overlap of trajectories in different electronic states.

References

(1) Bircher, M. P.; Liberatore, E.; Browning, N. J.; Brickel, S.; Hofmann, C.; Patoz, A.; Unke, O. T.; Zimmermann, T.; Chergui, M.; Hamm, P.; Keller, U.; Meuwly, M.; Woerner, H.-J.; Vaníček, J.; Rothlisberger, U. Nonadiabatic Effects in Electronic and Nuclear Dynamics. *Struct. Dyn.* **2017**, *4* (6), 061510. https://doi.org/10.1063/1.4996816.

(2) Martínez, T. J.; Levine, R. D. Non-Adiabatic Molecular Dynamics: Split-Operator Multiple Spawning with Applications to Photodissociation. *J. Chem. Soc. Faraday Trans.* **1997**, *93* (5), 941–947. https://doi.org/10.1039/A605958I.

(3) Makhov, D. V.; Glover, W. J.; Martinez, T. J.; Shalashilin, D. V. Ab Initio Multiple Cloning Algorithm for Quantum Nonadiabatic Molecular Dynamics. *J. Chem. Phys.* **2014**, *141* (5). https://doi.org/10.1063/1.4891530.
